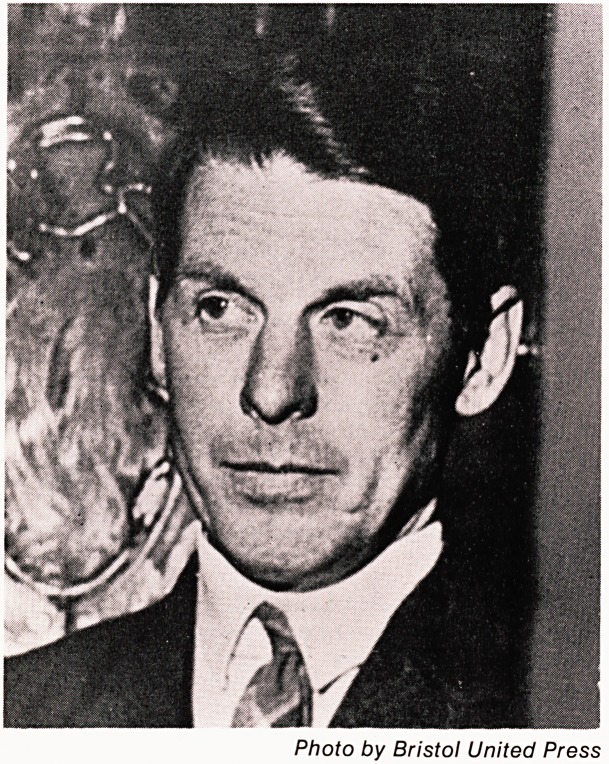# Charles H. Bartlett

**Published:** 1970-04

**Authors:** 


### Obituaries

### Charles H. BARTLETT, M.B., B.Chir., F.R.C.S.

*Photo by Bristol United Press*

Mr. C. H. Bartlett, consultant surgeon to Frenchay Hospital, Bristol, died on 4 October at the age of 55.

Charles Holmes Bartlett was born on 18 November 1913, and was educated at Warwick School and Christ's College, Cambridge. His long association with Bristol began when he joined the medical school for his clinical studies in 1935, graduating M.B., B.Chir. in 1938. He served in the R.N.V.R. during the second world war and after demobilization took the F.R.C.S. in 1946, and in 1947 returned to Bristol as surgical registrar to the new genito-urinary department of the Bristol Royal Infirmary. Charles Bartlett always retained a great interest in urology, but the needs of the service dictated his next move to Southmead Hospital, where, as senior surgical registrar, he did a great deal of emergency urological and general surgery. His steadiness, reliability, and capacity for sustained and concentrated work won him the deep respect of all his colleagues, senior and junior. It was natural, therefore, that when a department of general surgery at Frenchay Hospital was opened Charles Bartlett was selected as the first consultant surgeon. He retained his connexions with the university and with the teaching hospital through the work he did at the Homoeopathic Hospital.

Throughout his career Charles Bartlett remained the quiet, efficient, and reliable surgeon who was capable of getting through more work in a week than most men can do in two. He never wasted a word, rarely listened to small talk, and directed every ounce of his energy towards his work. He took a full part in hospital affairs but was shy of voicing an opinion unless asked to do so. However, his basic wisdom was such that his opinion was often sought not only in professional matters but also in those administrative decisions which an expanding department of surgery generated. He had a dry sense of humour which delighted those closest to him. To his immediate surgical colleagues he was a rock of common sense; to the physicians he was an utterly reliable surgeon whose decisions were good and whose patients thrived after surgery. To the anaesthetists, his junior staff, the nurses, and his patients he was the quiet hero. His care for, and kindness to, his patients were the hallmark of his career.

Three years ago, after he had been chairman of the Medical Advisory Committee at Frenchay Hospital for six months, he had his first attack of coronary thrombosis. He bore the enforced rest with patience, and later submitted to some easing of his work-load, but nothing could prevent Charles Bartlett from continuing to do more than his fair share of operating and patient-care. He took to sailing a small boat and some gentle gardening, and, as always, he had great joy and satisfaction in his growing family. He died after a short illness in the hospital for which he had done so much and where he will always be remembered for his complete integrity.

To his wife, Dr. Anne Bartlett, and his four children, who were such a comfort to him in his life, we extend our heartfelt sympathy.—T.J.B. and J.M.N.

## Figures and Tables

**Figure f1:**